# Souvenir of Bellevue Hospital Medical College

**Published:** 1871-01

**Authors:** 


### Souvenir of Bellevue Hospital Medical College.

Perhaps we shall be intruding into the sanctuary of private friendship if we tell, but it is nevertheless true, that Prof. James P. White, of Buffalo, has been made the recipient of a most elegant *Silver Epergne*, or *Table Centre Piece*, in token of appreciation of his recent services in Bellevue Hospital Medical College during the illness of Prof. Elliot.

It is truly in itseif beautiful; viewed as representing the spirit which prompted it, it is inestimable.

It bears the following inscription:

SOUVENIR
OF
BELLEVUE HOSPITAL MEDICAL COLLEGE.
1870.
PROFESSOR JAMES P. WHITE,
FROM HIS FRIEND
GEORGE Y. ELLIOT.

